# Supplementary material for: Association between endotypes of prematurity and pharmacological closure of patent ductus arteriosus: A systematic review and meta-analysis
Source: Front Pediatr. 2023 Mar 3;11:1078506. doi: 10.3389/fped.2023.1078506 (PMC10020634; doi:10.3389/fped.2023.1078506)
Supplement: Supplementary file 1 [file Datasheet1.pdf]

Supplementary Material

Association between endotypes of prematurity and pharmacological closure of patent ductus arteriosus: A systematic review and meta-analysis

Gema E Gonzalez-Luis[1], Moreyba Borges-Lujan[1] and Eduardo Villamor[2*]

[1]Department of Neonatology, Complejo Hospitalario Universitario Insular Materno-Infantil (CHUIMI) de Canarias, Las Palmas de Gran Canaria, Spain

[2]Department of Pediatrics, Maastricht University Medical Centre (MUMC+), School for Oncology and Developmental Biology (GROW), Maastricht, the Netherlands

*** Correspondence:**
Eduardo Villamor
e.villamor@mumc.nl

1 Supplementary Figures and Tables

1.1 Supplementary Figures

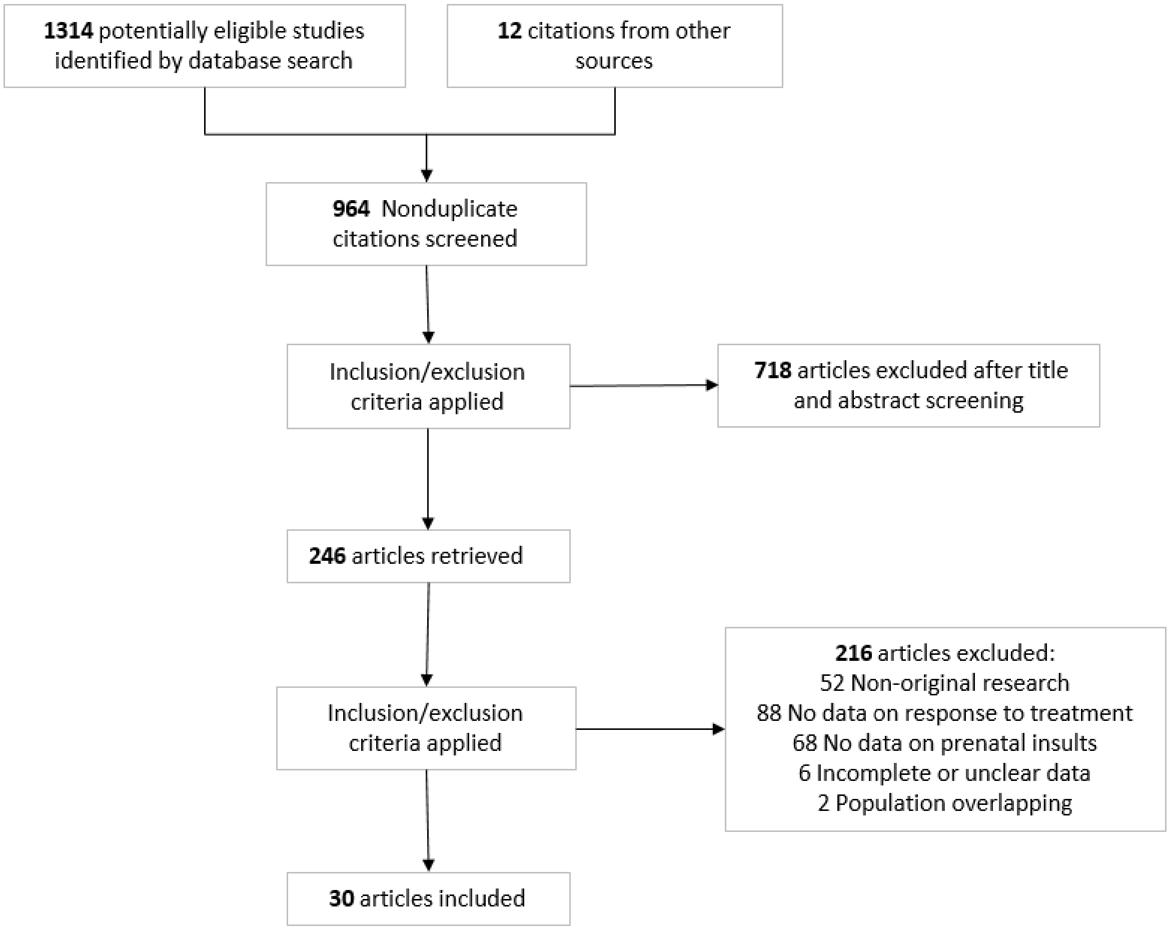

Supplementary Figure 1. Flow diagram of the systematic search

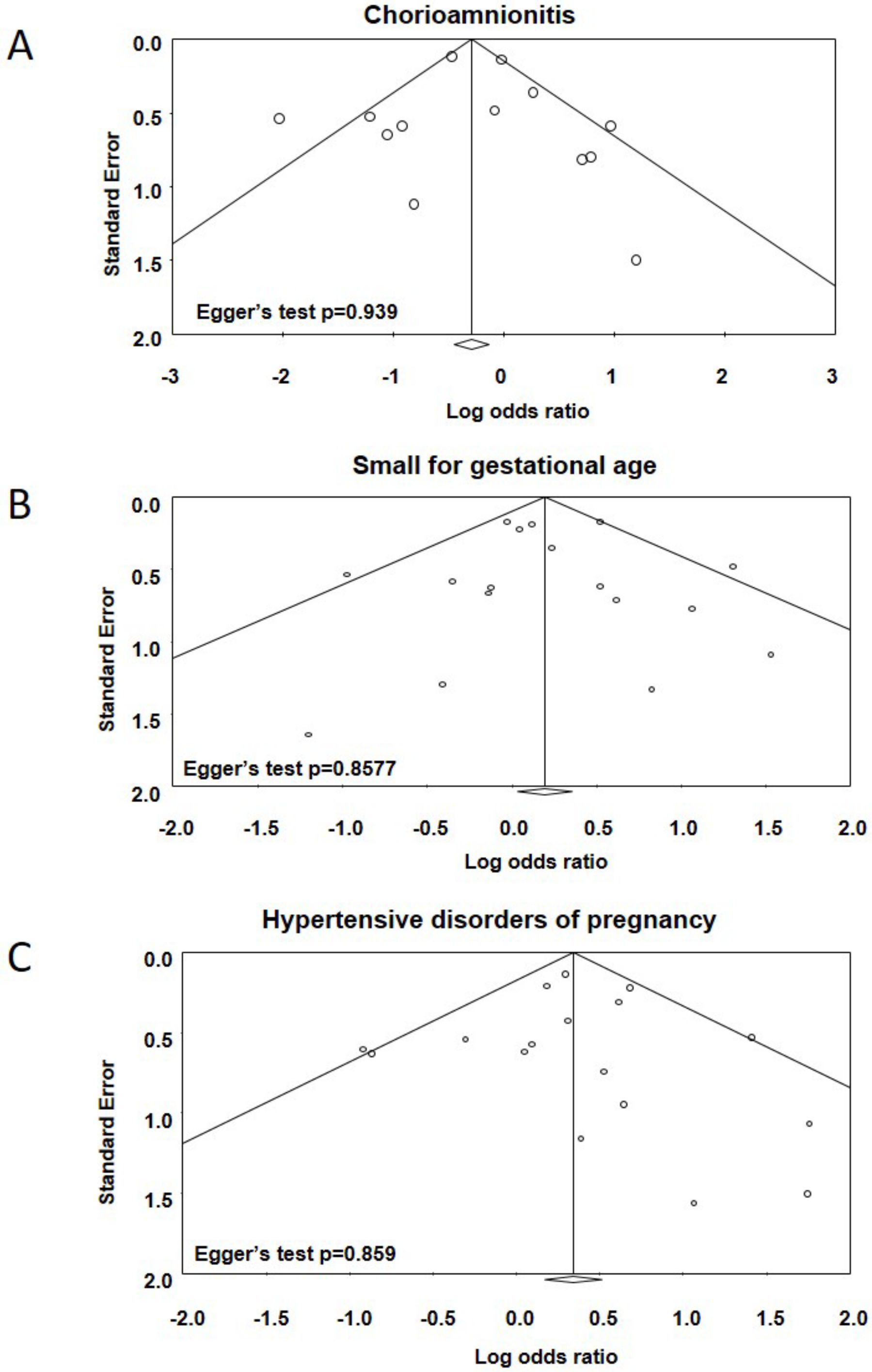

Supplementary Figure 2. Funnel plot for publication bias analysis for the studies included in the different meta-analyses.

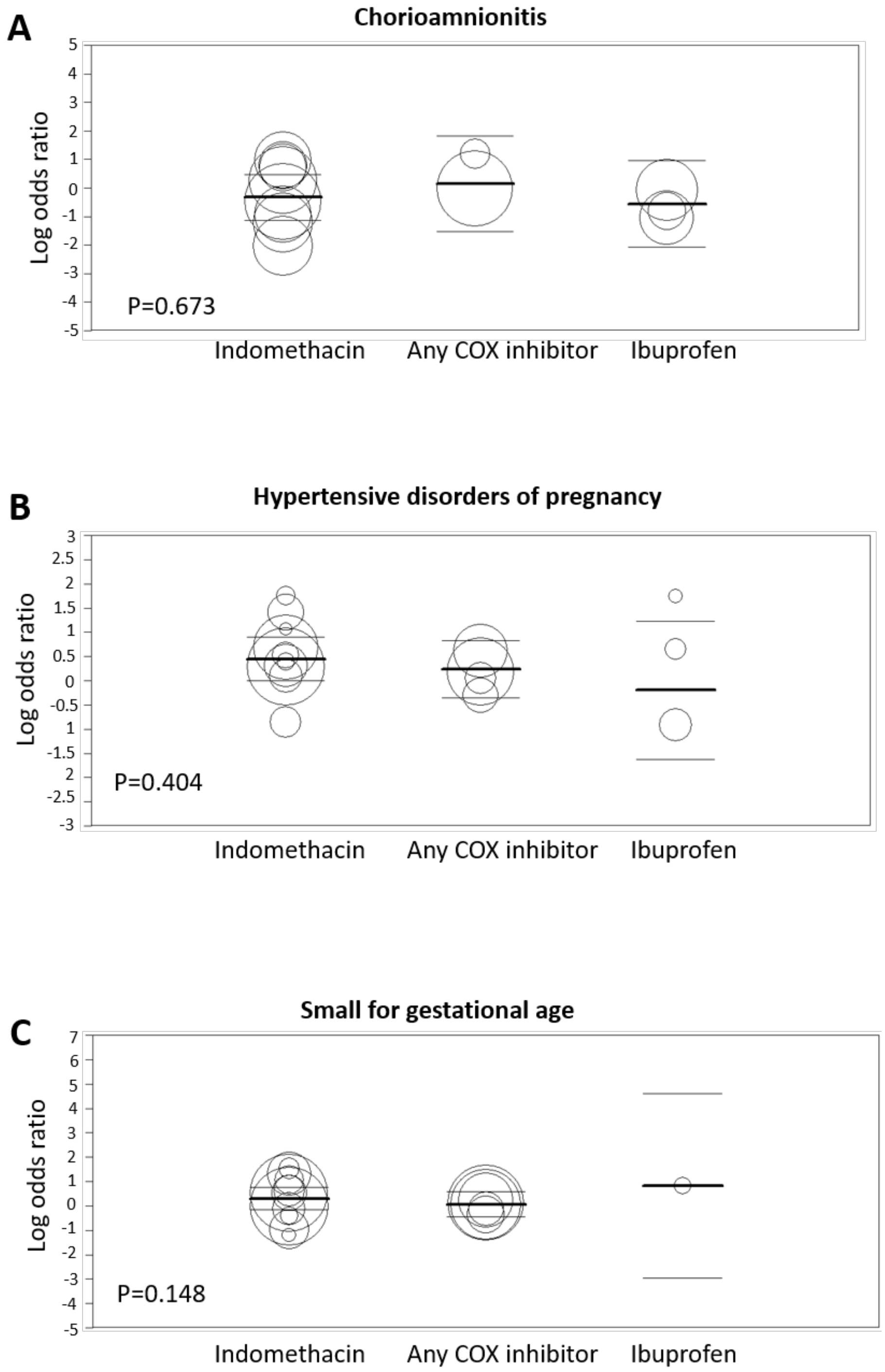

Supplementary Figure 3. Metaregression on the effect of the different cyclooxygenase (COX) inhibitors used in the studies.

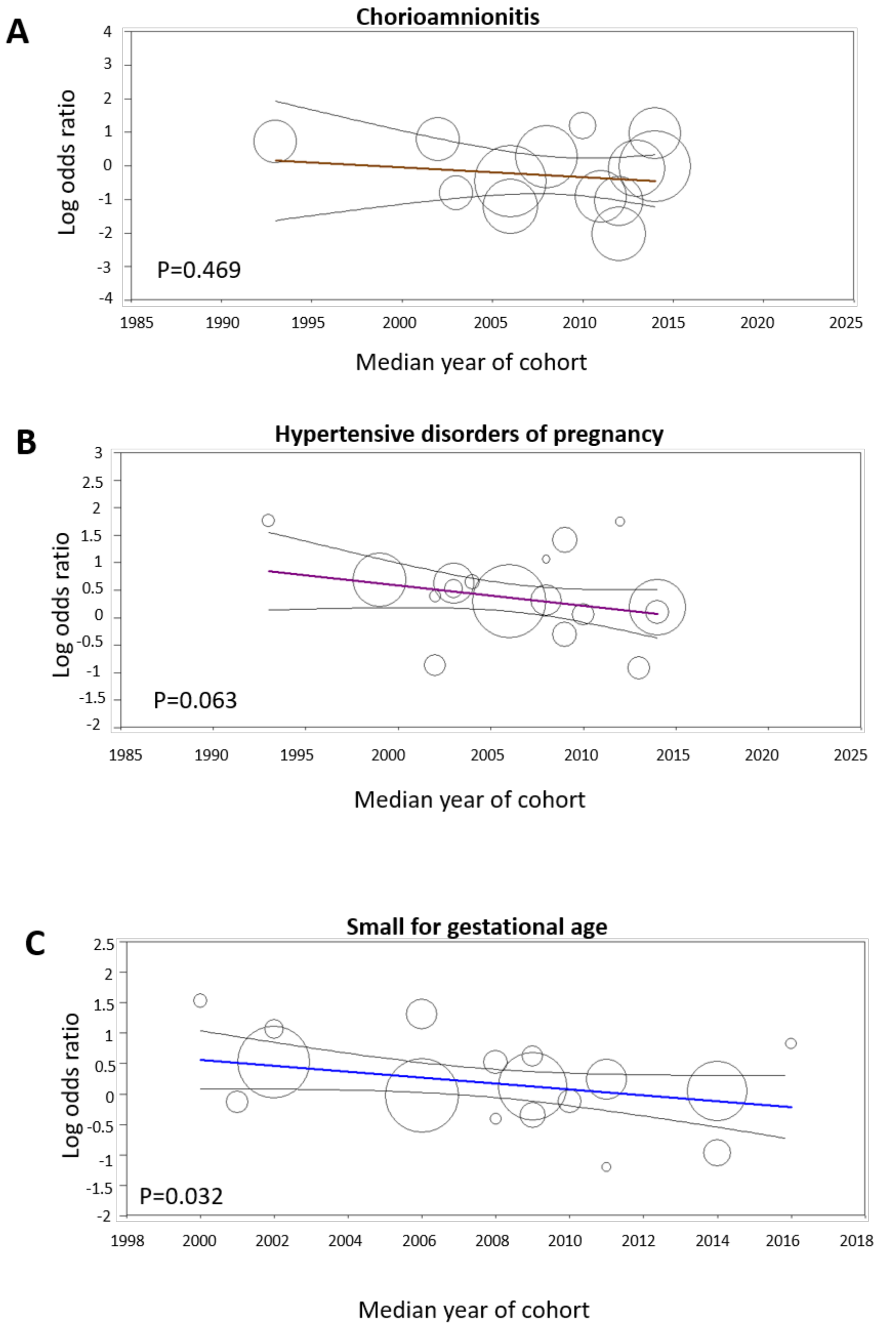

Supplementary Figure 4. Metaregression on the effect of the median year of the cohort in the different meta-analyses.

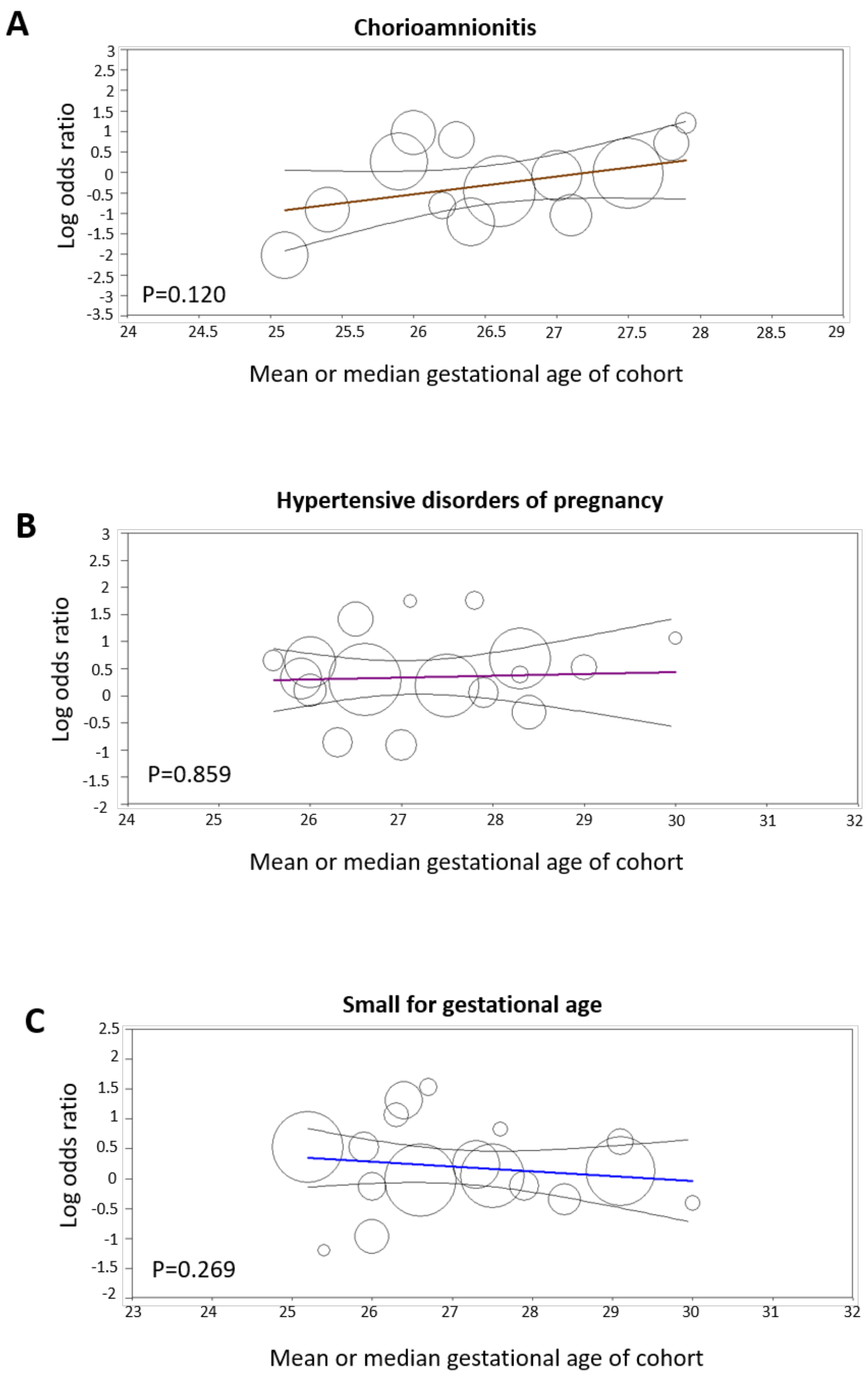

Supplementary Figure 5. Metaregression on the effect of gestational age of the cohort in the different meta-analyses.

Supplementary Table 1. Characteristics of the studies included in the meta-analyses.

First author. year	Country	Design	Prospective?	Total infants	Centers	GA (mean or median)	BW mean/median	Exposure	Treatment	Selection	Comparability	Outcome/ Exposure	Total NOS
Ahamed 2015 (1)	USA	Cohort	No	119	1	26.5	840.6	HDP	INDO	3	1	3	7
Bas 2014 (2)	Spain	Cohort	No	101	2	27.9	1008.5	Chorio /HDP/SGA	ANY	3	1	3	7
Boo 2006 (3)	Malaysia	Cohort	Yes	60	1	29.0	1115.8	HDP	INDO	3	2	3	8
Bravo 2011 (4)	Spain	Cohort	Yes	90	1	28.4	1066.7	HDP/SGA	ANY	4	2	3	9
Brooks 2005 (5)	Australia	Cohort	No	98	1	26.0	900.5	SGA	INDO	3	2	3	8
Dani 2008 (6)	Italy	Cohort	Yes	26	1	25.6	786.7	HDP	IBU	4	2	3	9
Dix 2016 (7)	Netherlands	Cohort	Yes	76	1	29.1	1312.4	SGA	INDO	3	1	3	7
Engeseth 2020 (8)	Germany	Cohort	Yes	91	1	26.7	865.4	SGA	INDO	3	1	3	7
Godambe 2006 (9)	Canada	Cohort	No	107	1	26.3	887.4	Chorio/HDP	INDO	3	2	3	8
Härkin 2018 (10)	Finland	Cohort	No	1132	Multi	29.1	1243.0	SGA	ANY	4	1	3	8
Hsu 2010 (11)	Taiwan	Cohort	Yes	31	1	30.0	1396.0	HDP/SGA	INDO	2	2	3	7
Hsu 2019 (12)	Taiwan	Cohort	No	18	1	27.6	1075.0	SGA	IBU	3	1	3	7
Itabashi 2003 (13)	Japan	Cohort	No	2508	Multi	28.3	1042.0	HDP	INDO	4	2	2	8
Kim 2010 (14)	Korea	Cohort	No	78	1	26.4	742.0	Chorio/SGA	INDO	2	2	3	7
Lee 2020 (15)	Korea	Cohort	No	1063	Multi	27.5	996.9	Chorio/HDP/SGA	ANY	4	2	3	9
Louis 2018 (16)	Canada	Cohort	No	98	1	25.4	772.0	SGA	INDO	3	1	3	7
Madan 2009 (17)	USA	Cohort	Yes	2435	Multi	25.2	736.2	SGA	INDO	4	2	2	8
Mirea 2012 (18)	Canada	Cohort	No	2652	22	26.6	ND	Chorio/HDP/SGA	INDO	3	2	3	8
Mitra 2015 (19)	Canada	Cohort	No	77	1	25.1	840.7	Chorio	INDO	3	2	2	7
Mydam 2019 (20)	USA	Cohort	No	91	1	25.4	827.9	Chorio	INDO	3	2	2	7
Oh 2020 (21)	Korea	Cohort	No	92	1	27.0	939.9	Chorio/HDP	IBU	3	2	3	8
Pees 2010 (22)	Germany	Cohort	Yes	15	1	26.2	853.8	Chorio	IBU	2	2	3	7
Rooney 2019 (23)	USA	Cohort	Yes	133	1	26.0	ND	Chorio/HDP/SGA	INDO	2	2	3	7
Sadeck 2014 (24)	Brazil	Cohort	No	307	16	27.3	787.1	SGA	ANY	3	1	3	7
Sallmon 2018 (25)	Germany	Cohort	No	471	2	26.0	867.0	HDP	ANY	3	2	3	8
Seon 2013 (26)	Korea	Cohort	No	58	1	27.1	946.0	Chorio/HDP	IBU	3	1	2	6
Shah 2011 (27)	USA	Cohort	Yes	397	1	25.9	828.2	Chorio /HDP/SGA	INDO	3	2	3	8
Uchiyama 2011 (28)	Japan	Cohort	No	57	1	28.3	1040.6	HDP	INDO	2	2	3	7
Weiss 1995 (29)	USA	Cohort	Yes	77	1	27.8	1076.6	Chorio/HDP	INDO	3	1	3	7
Yang 2008 (30)	Singapore	Cohort	No	40	1	26.3	807.3	SGA	INDO	3	1	3	7

BW: birth weight, Chorio: chorioamnionitis; GA: gestational age, HDP: hypertensive disorders of pregnancy; IBU: ibuprofen, INDO: indomethacin, SGA: small for gestational age.

References

1. Ahamed M, Verma P, Lee S, Vega M, Wang D, Kim M, et al. Predictors of successful closure of patent ductus arteriosus with indomethacin. Journal of Perinatology. 2015;35(9):729-34.

2. Bas-Suárez MP, González-Luis GE, Saavedra P, Villamor E. Platelet counts in the first seven days of life and patent ductus arteriosus in preterm very low-birth-weight infants. Neonatology. 2014;106(3):188-94.

3. Boo NY, Mohd-Amin I, Bilkis A, Yong-Junina F. Predictors of failed closure of patent ductus arteriosus with indomethacin. Singapore medical journal. 2006;47(9):763.

4. Bravo Laguna MC. Evaluación del tratamiento farmacológico convencional para el cierre del ductus arterioso persistencte en el recién nacido pretérmino: impacto de nuevas líneas terapéuticas. 2011.

5. Brooks J, Travadi J, Patole S, Doherty D, Simmer K. Is surgical ligation of patent ductus arteriosus necessary? The Western Australian experience of conservative management. Archives of Disease in Childhood-Fetal and Neonatal Edition. 2005;90(3):F235-FF9.

6. Dani C, Bertini G, Corsini I, Elia S, Vangi V, Pratesi S, et al. The fate of ductus arteriosus in infants at 23–27 weeks of gestation: from spontaneous closure to ibuprofen resistance. Acta Paediatrica. 2008;97(9):1176-80.

7. Dix L, Molenschot M, Breur J, de Vries W, Vijlbrief D, Groenendaal F, et al. Cerebral oxygenation and echocardiographic parameters in preterm neonates with a patent ductus arteriosus: an observational study. Archives of Disease in Childhood-Fetal and Neonatal Edition. 2016;101(6):F520-F6.

8. Engeseth MS, Engan M, Clemm H, Vollsæter M, Nilsen RM, Markestad T, et al. Voice and Exercise Related Respiratory Symptoms in Extremely Preterm Born Children After Neonatal Patent Ductus Arteriosus. Frontiers in pediatrics. 2020;8:150.

9. Godambe S, Newby B, Shah V, Shah PS. Effect of indomethacin on closure of ductus arteriosus in very-low-birthweight neonates 1. Acta Pædiatrica. 2006;95(11):1389-93.

10. Härkin P, Marttila R, Pokka T, Saarela T, Hallman M. Morbidities associated with patent ductus arteriosus in preterm infants. Nationwide cohort study. The Journal of Maternal-Fetal & Neonatal Medicine. 2018;31(19):2576-83.

11. Hsu J-H, Yang S-N, Chen H-L, Tseng H-I, Dai Z-K, Wu J-R. B-type natriuretic peptide predicts responses to indomethacin in premature neonates with patent ductus arteriosus. The Journal of pediatrics. 2010;157(1):79-84.

12. Hsu K-H, Wu T-W, Wu I-H, Lai M-Y, Hsu S-Y, Huang H-W, et al. Baseline cardiac output and its alterations during ibuprofen treatment for patent ductus arteriosus in preterm infants. BMC pediatrics. 2019;19(1):1-8.

13. Itabashi K, Ohno T, Nishida H. Indomethacin responsiveness of patent ductus arteriosus and renal abnormalities in preterm infants treated with indomethacin. The Journal of pediatrics. 2003;143(2):203-7.

14. Kim ES, Kim E-K, Choi CW, Kim H-S, Kim BI, Choi J-H, et al. Intrauterine inflammation as a risk factor for persistent ductus arteriosus patency after cyclooxygenase inhibition in extremely low birth weight infants. The Journal of pediatrics. 2010;157(5):745-50. e1.

15. Lee JA, Sohn JA, Oh S, Choi BM. Perinatal risk factors of symptomatic preterm patent ductus arteriosus and secondary ligation. Pediatrics & Neonatology. 2020;61(4):439-46.

16. Louis D, Wong C, Ye XY, McNamara PJ, Jain A. Factors associated with non-response to second course indomethacin for PDA treatment in preterm neonates. The Journal of Maternal-Fetal & Neonatal Medicine. 2018;31(11):1407-11.

17. Madan JC, Kendrick D, Hagadorn JI, Frantz III ID, Health NIoC, Network HDNR. Patent ductus arteriosus therapy: impact on neonatal and 18-month outcome. Pediatrics. 2009;123(2):674.

18. Mirea L, Sankaran K, Seshia M, Ohlsson A, Allen AC, Aziz K, et al. Treatment of patent ductus arteriosus and neonatal mortality/morbidities: adjustment for treatment selection bias. The Journal of pediatrics. 2012;161(4):689-94. e1.

19. Mitra S, Wahab MGA. Indomethacin dose-interruption and maternal chorioamnionitis are risk factors for indomethacin treatment failure in preterm infants with patent ductus arteriosus. Journal of Clinical Neonatology. 2015;4(4):250.

20. Mydam J, Rastogi A, Naheed ZJ. Base excess and hematocrit predict response to indomethacin in very low birth weight infants with patent ductus arteriosus. Italian journal of pediatrics. 2019;45(1):1-9.

21. Oh SH, Lee BS, Jung E, Oh MY, Do H-J, Kim EA-R, et al. Plasma B-type natriuretic peptide cannot predict treatment response to ibuprofen in preterm infants with patent ductus arteriosus. Scientific reports. 2020;10(1):1-7.

22. Pees C, Walch E, Obladen M, Koehne P. Echocardiography predicts closure of patent ductus arteriosus in response to ibuprofen in infants less than 28 week gestational age. Early Hum Dev. 2010;86(8):503-8.

23. Rooney SR, Shelton EL, Aka I, Shaffer CM, Clyman RI, Dagle JM, et al. CYP2C9* 2 is associated with indomethacin treatment failure for patent ductus arteriosus. Pharmacogenomics. 2019;20(13):939-46.

24. Sadeck LS, Leone CR, Procianoy RS, Guinsburg R, Marba S, Martinez FE, et al. Effects of therapeutic approach on the neonatal evolution of very low birth weight infants with patent ductus arteriosus. Jornal de pediatria. 2014;90(6):616-23.

25. Sallmon H, Weber SC, Dirks J, Schiffer T, Klippstein T, Stein A, et al. Association between platelet counts before and during pharmacological therapy for patent ductus arteriosus and treatment failure in preterm infants. Frontiers in pediatrics. 2018;6:41.

26. Seon H-S, Lee J-B, Kim I-U, Kim S-H, Lee J-H, Kim D-H, et al. Association with ductus arteriosus closure by ibuprofen and intrauterine inflammation in very low birth weight infants. Korean Journal of Perinatology. 2013;24(3):158-67.

27. Shah NA, Hills NK, Waleh N, McCurnin D, Seidner S, Chemtob S, et al. Relationship between circulating platelet counts and ductus arteriosus patency after indomethacin treatment. J Pediatr. 2011;158(6):919-23 e1-2.

28. Uchiyama A, Nagasawa H, Yamamoto Y, Tatebayashi K, Suzuki H, Yamada K, et al. Clinical aspects of very-low-birthweight infants showing reopening of ductus arteriosus. Pediatrics International. 2011;53(3):322-7.

29. Weiss H, Cooper B, Brook M, Schlueter M, Clyman R. Factors determining reopening of the ductus arteriosus after successful clinical closure with indomethacin. The Journal of pediatrics. 1995;127(3):466-71.

30. Yang C-Z, Lee J. Factors affecting successful closure of hemodynamically significant patent ductus arteriosus with indomethacin in extremely low birth weight infants. World Journal of Pediatrics. 2008;4(2):91-6.
